# The effect of obstructive sleep apnea and treatment with continuous positive airway pressure on stroke rehabilitation: rationale, design and methods of the TOROS study

**DOI:** 10.1186/1471-2377-14-36

**Published:** 2014-02-25

**Authors:** Justine A Aaronson, Coen AM van Bennekom, Winni F Hofman, Tijs van Bezeij, Joost G van den Aardweg, Erny Groet, Wytske A Kylstra, Ben A Schmand

**Affiliations:** 1 Heliomare Research & Development, Relweg 51, 1949 EC Wijk aan Zee, The Netherlands; 2 Department of Neurology, Academic Medical Center, University of Amsterdam, Meibergdreef 9, 1105 AZ Amsterdam, The Netherlands; 3 Department of Psychology, University of Amsterdam, Weesperplein 4, 1018 XA Amsterdam, The Netherlands; 4 Department of Pulmonary Medicine, Medical Centre Alkmaar, Wilhelminalaan 12, 1815 JD Alkmaar, The Netherlands; 5 Heliomare, PO Box 78 1940 AB, Beverwijk, The Netherlands

**Keywords:** Stroke, Rehabilitation outcome, Obstructive sleep apnea, CPAP, Randomized controlled trial, Cognition, Functional status

## Abstract

**Background:**

Obstructive sleep apnea is a common sleep disorder in stroke patients. Obstructive sleep apnea is associated with stroke severity and poor functional outcome. Continuous positive airway pressure seems to improve functional recovery in stroke rehabilitation. To date, the effect of continuous positive airway pressure on cognitive functioning in stroke patients is not well established. The current study will investigate the effectiveness of continuous positive airway pressure on both cognitive and functional outcomes in stroke patients with obstructive sleep apnea.

**Methods/Design:**

A randomized controlled trial will be conducted on the neurorehabilitation unit of Heliomare, a rehabilitation center in the Netherlands. Seventy stroke patients with obstructive sleep apnea will be randomly allocated to an intervention or control group (n = 2×35). The intervention will consist of four weeks of continuous positive airway pressure treatment. Patients allocated to the control group will receive four weeks of treatment as usual. Outcomes will be assessed at baseline, immediately after the intervention and at two-month follow-up.

In a supplementary study, these 70 patients with obstructive sleep apnea will be compared to 70 stroke patients without obstructive sleep apnea with respect to cognitive and functional status at rehabilitation admission. Additionally, the societal participation of both groups will be assessed at six months and one year after inclusion.

**Discussion:**

This study will provide novel information on the effects of obstructive sleep apnea and its treatment with continuous positive airway pressure on rehabilitation outcomes after stroke.

**Trial registration:**

Trial registration number: Dutch Trial Register NTR3412

## Background

Obstructive sleep apnea (OSA) is a sleep disorder characterized by repetitive cessations of breathing during sleep due to obstruction of the upper airway. The diagnosis of OSA is based on the mean number of apneas and hypopneas per hour sleep, the apnea-hypopnea index (AHI). Nighttime consequences of OSA are periodic oxygen desaturations, increases in blood pressure and sleep fragmentation. OSA is associated with an increased risk for cardiovascular diseases, such as hypertension, heart disease and stroke [1,2]. A recent meta-analysis [3] reported a prevalence of sleep apnea between 38-72% in stroke patients compared to 3-28% in the general population [4].

The most frequently reported daytime consequences of OSA are excessive daytime sleepiness and fatigue. There is also growing evidence that OSA negatively affects cognitive functioning and mood. Most studies find impairment in the cognitive domains of vigilance, attention, executive functioning, memory and motor coordination [5,6]. The etiology of the cognitive impairment in OSA patients is still unclear, but neuroimaging studies provide evidence that OSA is associated with structural and functional changes in the brain, particularly in the frontal cortex and hippocampus [7,8].

Continuous positive airway pressure (CPAP) is the treatment of choice for OSA. CPAP improves breathing during sleep, resulting in better blood oxygen saturation and less sleep fragmentation [9]. Consequently, it is expected that CPAP treatment will improve daytime functioning. However, evidence of the therapeutic effects of CPAP on fatigue, cognitive functioning and mood is inconsistent. A Cochrane review showed significant reduction of objective and subjective sleepiness, and depressive symptoms, while in a recent meta-analysis of cognitive functioning after CPAP we only found slight improvement in the attention domain [10,11]. Notwithstanding this limited evidence for behavioral effects of CPAP treatment, there are some indications that it results in structural changes in the brain. One imaging study showed that CPAP treatment was associated with increase of grey matter in the hippocampal and frontal structures [8].

OSA is a common sleep disorder in stroke and has been reported to be associated with stroke severity, poor functional outcome, recurrent stroke, and increased mortality [2,12-14]. In a cross-sectional study in stroke patients, OSA was found to be associated with delirium, depressed mood and impaired activities of daily living, but no relationship was seen between OSA and performance on a cognitive screening instrument (Mini Mental State Examination; MMSE) [15]. The authors suggested the use of more sensitive neuropsychological tests. We conducted a pilot study examining the effect of OSA on a set of neuropsychological tests in stroke patients [16]. In this small sample, OSA was found to be associated with lower performance on tasks of attention, verbal memory and visual scanning, and increased depressive symptoms.

To date, seven randomized controlled trials (RCTs) have compared CPAP to treatment as usual (TAU) or sham CPAP in stroke patients. Four of these studies found improvement in CPAP compared to TAU in one or more neurological function outcomes, sleepiness or mood [15,17-19], while three RCTs found no benefit of CPAP treatment in these areas compared to TAU or sham CPAP [20-22]. The latter studies had low compliance and insufficient power due to small sample size, which may have affected the results. The effect of CPAP on cognitive functioning was evaluated in three studies. Two studies used the MMSE [15,21] and one study [19] assessed the sustained attention to response test (a measure of vigilance) and the digit or spatial span backward test (a measure of executive function). None of these cognitive measures showed improvement with CPAP. The effect on more extensive neuropsychological assessment including sensitive measures of memory and attention has not yet been investigated.

In summary, previous research suggests that OSA is associated with poor functional outcome after stroke, and that treatment with CPAP improves functional recovery during stroke rehabilitation. However, the effect of OSA and CPAP treatment on cognitive functioning in stroke patients is still unclear. The Treatment of OSA and Rehabilitation Outcome in Stroke (TOROS) study is designed to address these issues.

The TOROS project consists of a randomized controlled trial (RCT), and a supplementary case–control study. The primary objective of the RCT is to evaluate whether CPAP treatment improves cognitive and functional outcomes in stroke patients with OSA (AHI ≥ 15), as assessed by a neuropsychological test battery and by measures of neurological status and functional dependence. The aim of the supplementary case–control study is to explore the association between OSA and cognitive and functional status in stroke patients. Our hypothesis is that stroke patients with OSA have a poorer functional status and greater cognitive impairment compared to stroke patients without OSA. In both the RCT and the case–control study the secondary objectives are: 1) to evaluate the effects of OSA (and its treatment) on sleep quality, fatigue and mood; and 2) to evaluate the long-term effects of OSA on societal participation.

## Methods

### Design

In the RCT, 70 OSA patients will be randomized to receive four weeks of CPAP treatment or four weeks of TAU. Patients randomized to TAU will start with CPAP treatment after the four-week intervention period. Neuropsychological assessment and examination of functional status, the primary outcome measures, are conducted at baseline and repeated after the four-week intervention period and at two-month follow-up. The secondary measures sleep quality, fatigue, and mood are assessed at the same time points as the primary outcome measures. To ascertain whether OSA improves with CPAP treatment, direct measurements of OSA, including AHI and level of oxygen desaturation during sleep, are made at baseline, at the end of the intervention period, and at two-month follow-up. To assess treatment compliance, the CPAP device registers CPAP usage per night, the air pressure and the residual AHI. At four weeks, and three, six and twelve months after enrollment the rehabilitation physician checks the compliance.

To test the hypothesis of the case–control study that OSA is associated with poor functional status and greater cognitive impairment, 70 stroke patients diagnosed with OSA are compared to 70 stroke patients without OSA on the primary and secondary outcomes at admission to the rehabilitation unit (baseline).

In a subgroup of the stroke patients without OSA (N = 35) the assessment of primary and secondary outcome measures will be repeated after four weeks and three months. The outcomes will be compared to the recovery of OSA patients.

**Figure 1:**
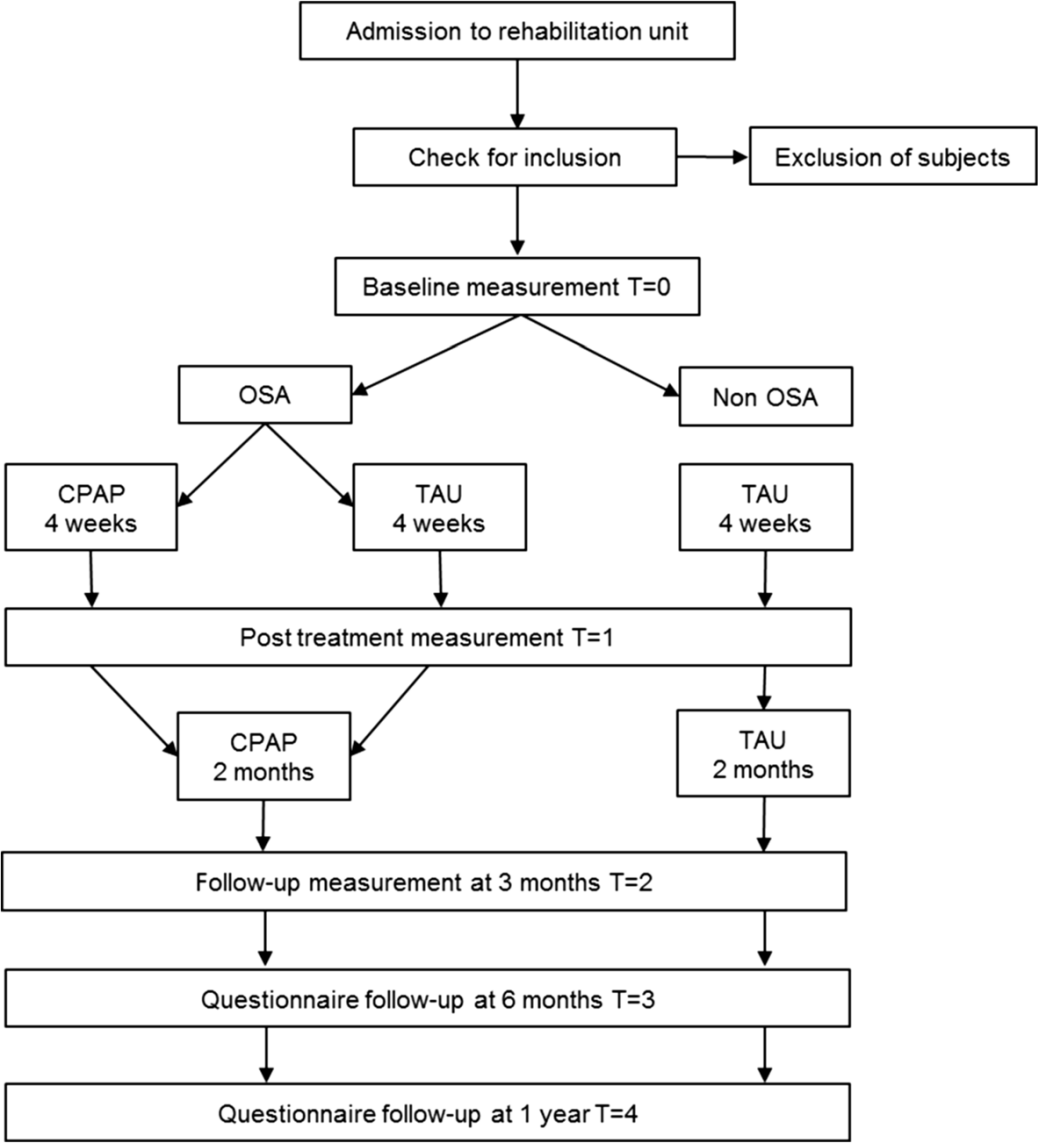
**Flowchart of the TOROS study.** OSA: obstructive sleep apnea; CPAP: continuous positive airway pressure; TAU: treatment as usual.

All patients included in the TOROS study are asked to fill out a questionnaire on participation in work, leisure and social activities at six months and one year after inclusion. The study design is illustrated in Figure 1.

The Medical Ethical Review Board of the Academic Medical Centre, Amsterdam, approved the study. The inclusion period started in October 2011. The study is registered at the Dutch trial register (http://www.trialregister.nl) and identified as NTR3412.

### Patient sample and procedures

This study is conducted in Heliomare rehabilitation center in the Netherlands. Annually, approximately 150 stroke patients are admitted to Heliomare. Neurologists or rehabilitation physicians of hospitals in the surrounding area refer patients. Patients with vascular dementia or comorbid neurodegenerative diseases (e.g., Alzheimer’s or Parkinson’s disease) are excluded from rehabilitation in Heliomare and are referred to the rehabilitation unit of a nursing home. The majority of the admitted patients are in the post-acute phase, i.e., on average, two to three weeks post-stroke. The average length of stay in Heliomare is nine weeks (standard deviation of four weeks). An earlier study showed that the prevalence of OSA in stroke patients admitted to Heliomare is around 50% [23]. At admission to the rehabilitation unit, stroke severity and functional dependence scales are used to determine the patient’s functional status. In the first week of hospitalization, patients are interviewed with a questionnaire to determine the presence of common symptoms of OSA. Subsequently, their oxygen desaturation index (ODI), defined as the mean number of desaturations of ≥3% from baseline per hour, is assessed with nocturnal pulse oximetry. Patients with an ODI of five or higher are further tested for OSA by polygraphy. Patients with an AHI of 15 or higher on polygraphy are diagnosed with OSA and patients with a normal ODI (<5) [23,24] or AHI below 15 are defined as non-OSA patients. Within four weeks of admission, a neuropsychological assessment is administered to determine the level of cognitive functioning. Stroke patients meeting the following inclusion criteria are invited to participate within six weeks from admission to Heliomare: [1] stroke confirmed by a neurologist, [2] age between 18 and 85 years, [3] admission to Heliomare between 1 and 16 weeks post-stroke, and [4] able to participate in the OSA screening and neuropsychological assessment. Exclusion criteria are: [1] severe unstable medical conditions, respiratory failure or history of severe congestive heart failure, [2] traumatic brain injury, [3] severe aphasia, confusion or psychiatric comorbidity, [4] central sleep apnea or previously diagnosed OSA. Patients with severe OSA (AHI >60 and oxygen desaturations below 70%), which could endanger the patient’s health if treatment is not immediately started, are excluded from the RCT part of the study. Before inclusion, patients give written informed consent.

### Randomization and blinding

Participants with OSA will be randomized immediately after the baseline assessments. The minimization technique [25] will be used to minimize imbalance between the treatment arms for age, severity of OSA, stroke subtype (ischemic or hemorrhage) and severity of cognitive impairment. Assessors of cognitive and functional outcome measures will be blinded to treatment allocation.

### Intervention

CPAP treatment is set up and monitored by a specialized ‘Respicare’ team. This team exists of two rehabilitation physicians, two nurse practitioners and four nurses working on the neurorehabilitation unit specialized in sleep and breathing disorders. Before treatment is initiated, a CPAP mask, connecting hose and CPAP device are set up for each patient. Different masks are used, from small nasal pillows to a full-face mask, depending on the patient’s preference. Personalized instructions are given by one of the Respicare team members and a written manual for the CPAP device is provided. If possible, the partner or a close relative is also provided with instructions on the use of the CPAP device. Patients are asked to wear the mask for a short period during the day to become accustomed to using the CPAP device. Within the first week, the CPAP treatment is evaluated together with the patient and CPAP titration is performed using pulse oximetry. The pressure is adjusted until the ODI is reduced to normal (ODI <5). If titration by nocturnal oximetry fails to adequately reduce the ODI, CPAP is titrated by polygraphy to reduce the AHI to <5 or to the highest pressure tolerated. The CPAP device is provided with a memory card to evaluate the effectiveness of CPAP therapy over time and to monitor CPAP compliance. The Respicare team has contact with the patients regularly during the intervention period to help troubleshoot problems and encourage compliance. Patients who are discharged during the four-week intervention period are followed-up by telephone.

### Primary outcomes

The primary outcome measures of this study are cognition and functional status. The following nine cognition domains are assessed: vigilance, attention, memory, working memory, executive functioning, language, visuoperception, psychomotor ability and intelligence. The neuropsychological test battery of standardized neuropsychological tests [26] is assessed by a trained psychological assistant. For a number of cognitive domains, non-verbal alternative tests are included for patients with aphasia. A language comprehension test is also included for aphasic patients. The obtained test scores are transformed into demographically corrected z-scores. In case of multiple tests within one domain, the average z-score for the domain is calculated. In Table 1 all tests are summarized by cognitive domain.

**Table 1:** Neuropsychological tests by cognitive domain

***Cognitive domain***	***Neuropsychological test***
Vigilance	Psychomotor vigilance task
Attention	D-Kefs trail making test (TMT)
	*Color trails test
	d2 Test of attention
Memory	Rey’s auditory verbal learning test
	*Location learning test
Working Memory	WAIS-III letter-number sequencing
	*WMS-IV symbol span
Executive functioning	D-Kefs TMT letter-number switching
	Tower of London
Language	GIT-II category fluency
	**Token test
Visuoperception	D-Kefs TMT visual scanning
	Bells test
Psychomotor ability	D-Kefs TMT motor speed
	Finger tapping
Intelligence	WAIS-III matrix reasoning

*non-verbal alternative; **only administered in patients with aphasia.

Functional status is assessed by measures of neurological status and functional dependence. The rehabilitation physician administers two scales of neurological status, the Canadian Neurological Scale [27] and the National Institutes of Health Stroke Scale [28]. The obtained scores are transformed into z-scores and averaged into one score for neurological status. The nurse practitioner scores the level of functional dependence on the Utrecht Scale for Evaluation of Rehabilitation [29]. This scale can be converted to the Barthel Index [30].

### Secondary outcomes

Secondary outcome measures of the TOROS study are sleepiness (Stanford Sleepiness Scale), fatigue (Checklist Individual Strength), mood (Hospital Anxiety and Depression Scale), and subjective sleep quality (Sleep Quality Scale) [31-34]. A trained psychological assistant administers these measures after the neuropsychological assessment. The Utrecht Scale for Evaluation of Rehabilitation – Participation [35] is used to evaluate work, leisure and social activities at six months and one year after the inclusion. The patients complete this questionnaire at home.

Objective measurements of sleep are made using standardized pulse oximetry (WristOx®; Nonin Medical, Plymouth, USA) and ambulatory overnight cardiorespiratory polygraphy (Embletta®; Embla, Ottowa, Canada). The ODI (mean number of oxygen desaturations of ≥3% per hour) is calculated from the pulse oximetry data using automated analysis. Trained staff manually scores the polygraph recordings. Apnea is defined as a reduction of airflow of ≥90% for at least 10 seconds and hypopnea is defined as a reduction of airflow of ≥50% for at least 10 seconds followed by an oxygen desaturation of ≥3%. The AHI is defined as the mean number of apneas and hypopneas per hour in bed. The change of AHI measured by polygraphy and treatment compliance are used as moderating variables in the RCT.

A self-report questionnaire is administered to identify OSA symptoms (e.g., snoring, daytime sleepiness, morning headaches). Socio-demographic and clinical characteristics such as age, sex, education, body mass index (BMI), medication use and life style variables (e.g., smoking) are obtained from medical records.

### Sample size

We conducted two separate power analyses for the RCT; one for the functional outcome and one for the cognitive outcome. The power calculation for the functional outcome is based on two previous studies [18,19]. These studies found large effects of CPAP treatment on functional outcome measures in stroke patients. To detect at least one standard deviation (large effect) on functional outcome with 90% power and an alpha of 0.05 (one-tailed), a minimum of 34 patients (17 per arm) is required.

No reliable information on the expected effect of CPAP treatment on cognitive measures is available. Therefore, an effect size of 0.75 standard deviation was used to estimate the necessary sample size. Taken the same alpha level and 80% power, a minimum of 56 patients (28 per arm) needs to be included in the RCT. The compliance in this study is expected to be relatively high (around 67%) due to the relatively short duration of the experimental treatment phase and the assistance of a specially trained staff. Taking the compliance rate into account, a total of 70 patients will be the target sample size for the RCT.

For the supplementary case–control study, we set the sample size at 70 OSA patients and 70 patients without OSA, which allows detection of a medium size effect (0.5 standard deviation) with 90% power, and p-value set at 0.5.

### Statistical analyses

All statistical analyses will be performed with SPSS 19 (IBM; Armonck, USA) or later versions of this package. Depending on the level of measurement, parametric statistics (Student’s *t*-test and analysis of (co)variance) or non-parametric statistics (*χ*^2^ and Fisher’s exact test) will be used. Statistical significance will be set at a p-value of 0.05 or less. Socio-demographic and clinical characteristics at baseline will be presented using descriptive statistics. If group differences are observed at baseline on one or more background variables, those variables will be included as covariates in all further analyses.

For the RCT, the therapy effects on the primary and secondary outcome measures will be examined using a two factor (group × time) multivariate analysis of variance (MANOVA). Effect sizes will be calculated for all analyses using standard statistical procedures. To control for the multiple comparisons of cognition and functional status, two separate MANOVA’s will be performed. Given that we have two primary outcomes defined for the study (i.e., cognitive and functional outcomes), we will subsequently follow Hochberg’s step-up procedure [36] to control the false discovery rate.

Data will be analyzed according to the intention-to-treat principle. Additionally, a per-protocol analysis will be performed. Depending on the variability of compliance, a secondary analysis of good versus poor compliance will be conducted. In an exploratory analysis, we will investigate whether the effectiveness of CPAP therapy varies as a function of clinical diagnosis, level of cognitive functioning, and demographic background.

For the supplementary case–control part of the study, OSA patients will be compared with the control group of non-OSA patients on primary and secondary outcome measures using a MANOVA. As with the RCT, a separate analysis will be performed for cognition and functional status, and the Hochberg’s step-up procedure will be followed. For the follow-up of the USER-P questionnaire, within group changes will be assessed using paired t-tests and between group comparison will be made with two sample *t*-test.

## Discussion

Sleep apnea is highly prevalent in stroke patients. The TOROS study is designed primarily to evaluate the effect of CPAP treatment on improvement of rehabilitation outcome in stroke patients. In the RCT part of the study, conducted in stroke patients with OSA, CPAP treatment is compared to TAU on both functional and cognitive outcome. There is growing evidence that CPAP improves functional status [17-19], but the effect of CPAP on cognitive functioning in stroke patients with OSA remains unclear. CPAP treatment of OSA patients who did not suffer stroke has only a limited effect on cognitive functioning [11]. However, stroke patients with OSA have much more to gain with respect to cognitive functioning than these ‘regular’ OSA patients. Thus, the cognitive effects of CPAP treatment may be much larger in stroke patients with OSA.

Treatment compliance is a major practical problem with CPAP. A number of earlier studies investigating the effects of CPAP treatment suffered from low compliance [20-22]. To ensure treatment compliance within this project, a specialized Respicare team will carefully monitor patients receiving CPAP treatment in order to quickly detect and resolve problems, and motivate patients to continue use of the treatment. We expect that this will enhance treatment compliance.

The supplementary case–control study will improve our understanding of the association between OSA and cognitive impairments in the rehabilitation of stroke patients. Previous studies provide provisional evidence that OSA in stroke patients is associated with poor functional outcome [13], but the association between OSA and cognitive functioning in stroke patients is far from clear. Studies conducted in regular sleep clinics found that OSA negatively affected several cognitive domains [5,6]. Therefore, it is expected that OSA in stroke patients is associated with poor cognitive outcome.

There are several limitations to the TOROS study that should be noted. Firstly, we perform polygraphy rather than polysomnography to diagnose OSA. Our experience is that polygraphy is often better tolerated by stroke patients, but polygraphy has the disadvantage that it may give an underestimation of the AHI in case of low sleep efficiency. Other limitations of the study are the relatively small sample size and short CPAP intervention period. These are practical limitations that cannot be overcome given both financial and time constraints in carrying out this research.

## Conclusions

To date, there are no guidelines for the screening and treatment of OSA in stroke rehabilitation. We believe that the TOROS study will add to the understanding of the clinical implications of OSA in stroke rehabilitation, and the effects of CPAP treatment on the rehabilitation outcome. This, in turn, will provide rehabilitation physicians with evidence to help formulate future guidelines for OSA in stroke patients.

## Abbreviations

AHI: Apnea-hypopnea index; CPAP: Continuous positive airway pressure; MMSE: Mini mental state examination; ODI: Oxygen desaturation index; OSA: Obstructive sleep apnea; RCT: Randomized controlled trial; TAU: Treatment as usual; TOROS: Treatment of obstructive sleep apnea and rehabilitation outcome in stroke patients.

## Competing interests

The authors declare that they have no competing interests.

## Authors’ contributions

CvB, TvB, EG and WK conceived the idea of this study. JA, CvB, WH, TvB, JvdA, EG, WK contributed to the design of the study. JA coordinates the study under supervision of BS, CvB and WH. TvB enables recruitment and monitors the intervention treatment. JA was the primary author for this manuscript. BS helped draft the manuscript. All authors critically reviewed the manuscript and approved the submitted version.

## Pre-publication history

The pre-publication history for this paper can be accessed here:

http://www.biomedcentral.com/1471-2377/14/36/prepub
